# Mechanical Effects of Wrist Position at the Wrist Joint: A Finite Element Analysis

**DOI:** 10.1016/j.jhsg.2025.100747

**Published:** 2025-05-23

**Authors:** Takashi Nomoto, Yusuke Matsuura, Takahiro Yamazaki, Takane Suzuki, Seiji Ohtori

**Affiliations:** ∗ Department of Orthopaedic Surgery, Graduate School of Medicine, Chiba University, Chiba, Chiba Perfecture, Japan; † Department of Bioenvironmental Medicine, Graduate School of Medicine, Chiba University, Chiba, Chiba Perfecture, Japan

**Keywords:** Finite element analysis, Fresh-frozen cadavers, Stress distribution, Wrist joint

## Abstract

**Purpose:**

This study aimed to evaluate the effects of wrist joint angles during flexion–extension and radioulnar deviation on stress distribution using finite element analysis.

**Methods:**

Eight fresh-frozen upper limb specimens were analyzed using computed tomography. Finite element models were developed to simulate grip postures in flexion–extension (five positions ranging from 30° flexion to 30° extension) and radioulnar deviation (eight positions from 15° radial deviation to 20° ulnar deviation). Stress distributions (equivalent stress, minimum principal stress, and maximum principal stress) in the distal radius, ulnar head, and proximal carpal bones were assessed.

**Results:**

In the flexion–extension model, stress was concentrated in the central area of the distal radius and increased with an increase in flexion–extension angles. Stress values in the ulnar head and triquetrum increased during flexion and extension, whereas stress changes were minimal in the scaphoid and lunate. The scaphoid fossa experienced higher stress than the lunate fossa, with the volar aspect of the distal radius under greater stress during extension and the dorsal aspect during flexion. In the radioulnar deviation model, radial deviation decreased the load on the lunate fossa while increasing the load on the ulnar head, triquetrum, and dorsal lunate. Conversely, ulnar deviation reduced the load on the ulnar head but increased the load on the volar aspect of the lunate fossa.

**Conclusions:**

Finite element analysis demonstrated dynamic changes in wrist joint stress distribution at various motion angles.

**Clinical relevance:**

These findings enhance the understanding of wrist biomechanics and provide insights into the pathomechanics of degenerative wrist conditions.

The wrist joint is a complex structure vital for performing activities of daily living. It comprises eight carpal bones that articulate with the radius and ulna, enabling a wide range of motion and precise movement while bearing significant mechanical loads.

Numerous studies have explored the loading dynamics of the wrist joint. Research has revealed that the wrist experiences considerable compressive and shear forces during activities ranging from routine tasks to forceful gripping.^1^^,^^2^ Notably, gripping activities transmit forces through the wrist joint that exceed initial expectations.^3^

Although load transmission and distribution within the wrist joint have been studied extensively, uncertainties remain regarding how changes in carpal bone alignment during flexion–extension and radioulnar deviation influence load distribution across the midcarpal and radiocarpal joints.^4^, ^5^, ^6^, ^7^, ^8^, ^9^

Previous biomechanical studies have been limited by the potential for measurement devices to disrupt ligaments and soft tissues, altering natural joint mechanics. Finite element analysis (FEA) has emerged as a reliable tool for biomechanical assessment. Finite element analysis is a computational simulation used to predict internal forces within an object. When an external force is applied, objects undergo deformation. To model this behavior, an object is discretized into small elements. By calculating the forces acting on each element and their resulting deformations, followed by combining these interactions, FEA enables the prediction of stress and strain distributions across the entire structure. Finite element analysis enables the analysis of stress distribution without compromising tissue integrity and has proven successful in clinical applications, including fracture prediction and other clinical areas.^10^ This study aimed to evaluate stress distribution across different wrist positions using FEA and examine how changes in joint angles affect stress distribution.

## Materials and Methods

### Specimen

We obtained 10 upper limbs from 10 fresh-frozen cadavers from our university’s clinical anatomy laboratory. The donors were five men and five women. The exclusion criteria were a history of surgery on the forearm or hand, trauma, osteoarthritis, or ulnar variance of ≥ 2 mm. Thus, two upper limbs were excluded, and eight upper limbs (five right upper limbs and three left upper limbs) were subjected to further analysis. The mean age at death was 89.79 ± 7.32 years (range: 74–98 years).

The specimens were obtained fresh and frozen at −22 °C. The specimen was cut in the middle of the upper arm and thawed at room temperature immediately before computed tomography (CT) imaging. The area beyond the cut was not cleaned of soft tissue, and the cut surface was covered with gauze moistened with saline solution to prevent drying. No specimens were refrozen.

### Modeling conditions

Computed tomography was performed using Aquilion ONE (Toshiba Medical Systems) with the following imaging parameters: 320-row detector; 120 kV; 200 mA; 0.63-mm slice thickness; and 0.5-mm pixel width. A calibration phantom (QRM-BDC, QRM, MÖhrendorf, DE) containing three hydroxyapatite rods (0, 100, and 200 mg/cm^3^) was taken with the specimens. The specimens were fixed in the neutral forearm position on the CT table, and the scanning range extended from the midhumerus distally.

To create models that simulate grip movement in the flexion–extension positions (flexion–extension model), we obtained scans at five different angles, ranging from 30° flexion (FL) to 30° extension (EX) at 15° intervals, with 0° radioulnar deviation. To create models that simulate grip movement in the radioulnar positions (radioulnar deviation model), we placed the palmar aspect horizontally on the table in the grip position and obtained scans at eight different angles, ranging from 15° radial deviation (RD) to 20° ulnar deviation (UD) at 5° intervals. We measured the wrist joint angle using a goniometer, measuring the longitudinal axis of the third metacarpal and midline of the forearm. Three-dimensional (3D) models were created from the CT data and evaluated by FEA using MECHANICAL FINDER (Research Center of Computational Mechanics, Inc).

### FE modeling

**Figure 1:**
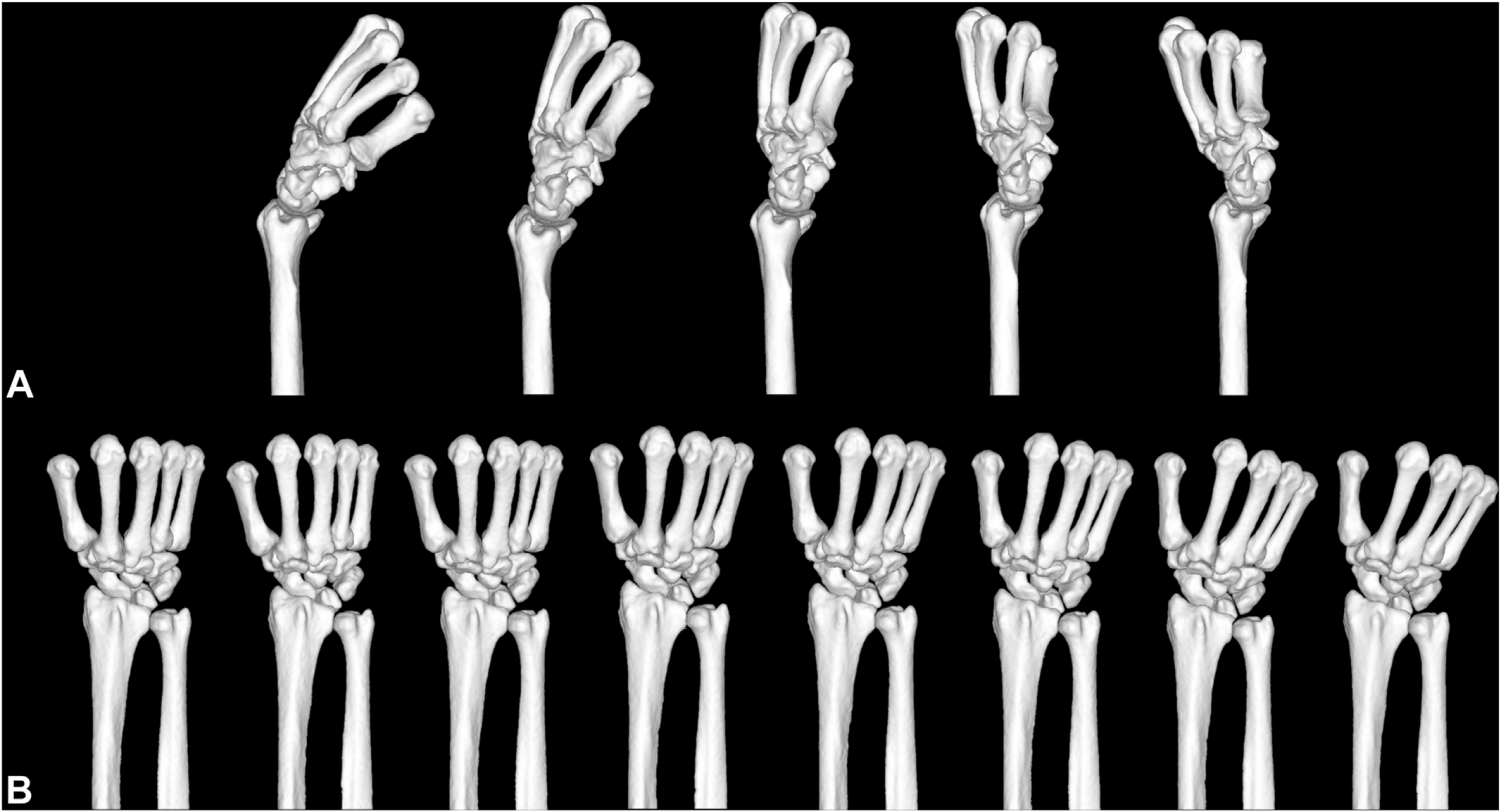
Finite element models of different wrist positions. **A** Models with 30° flexion to 30° extension. **B** Models with 15° RD to 20° UD. Thirteen models were created from each cadaveric specimen (total: 104 models), covering the region from the midforearm to the metacarpals.

Thirteen models were created from the CT data of a cadaveric specimen, and a total of 104 models were created using eight cadaveric specimens (Fig. 1). The region of interest (ROI) included the bones from the middle portion of the radius and ulna to the metacarpals, with all soft tissues removed. The 3D models were meshed into 1-mm thick tetrahedral elements, and the surface was covered with 1-mm thick shell elements.^11^^,^^12^ The imaginary thickness of the shell element was set to 0.3 mm. Computed tomography values were converted into bone mineral density using the following formula:

Bonemineraldensity,ρ=a×CTvalue(Hounsfieldunitvalue)+b

where a and b are the correction values obtained using calibration phantom.

The bone mineral density value, ρ obtained from the CT data, was converted into Young’s modulus E (MPa) using the formulas reported by Matsuyama based on a previous validation study.^12^

$$E\;\left(\text{MPa}\right)=1530.6\;\sigma^{1.9213}\left(\sigma \;>\;0\right),E=0.001\left(\sigma =0\right)$$

**Figure 2:**
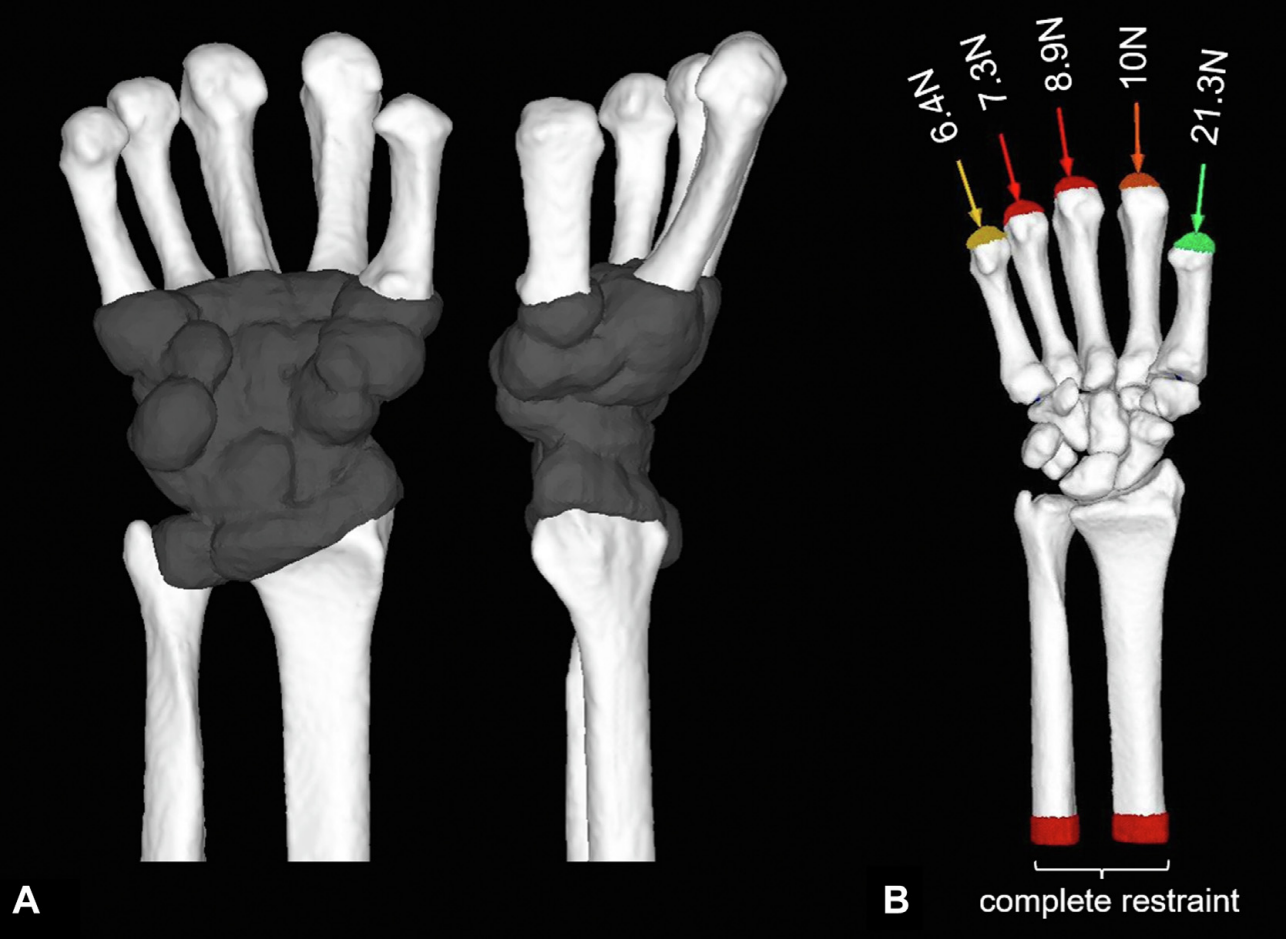
Wrist joint modeling and boundary conditions for FEA. **A** After cartilage modeling. **B**. Boundary conditions. Cartilage regions were established between the bones. The ROIs of the proximal metacarpals, carpal bones, distal radius, and ulnar head were expanded by 2 mm and replaced with cartilage. The middle portion of the radius and ulna shaft was fully constrained, and different loads were applied along the longitudinal axis of each metacarpal (thumb: 21.3 N, index: 10 N, middle: 8.9 N, ring: 7.3 N, little: 6.4 N; total: 53.9 N).

The Poisson’s ratio was set at 0.4.^13^^,^^14^ The cartilage regions were established between the bones. The ROIs of the proximal metacarpals, carpal bones, distal radius, and distal ulna were expanded by 2 mm and replaced with cartilage to fill all interosseous spaces (Fig. 2A).^11^ The Young’s modulus of the cartilage was set at 10 MPa, and the Poisson’s ratio was set at 0.49.^11^^,^^13^ The yield stress of the cartilage elements was set sufficiently high to prevent failure.

### Boundary conditions

Contact analysis was performed at the boundaries between the cartilage and the distal radius and ulna. A frictionless contact (friction coefficient μ = 0) was used to avoid the generation of shear stress in the joints.^15^ The contact between cartilage and bone was defined as bonded, except for the contact analysis regions. The midradius and ulnar shafts were fully constrained, whereas all other regions were not constrained in terms of displacement and rotation in all directions. Based on previous biomechanical studies that simulated grip loading,^16^ different loads were applied along the longitudinal axis of each metacarpal (thumb: 21.3 N, index: 10 N, middle: 8.9 N, ring: 7.3 N, little: 6.4 N; total: 53.9 N; Fig. 2B).^17^, ^18^, ^19^ The loading condition simulates 1/24 of the maximum gripping force for a healthy individual.^16^

### Evaluation

#### Stress distribution in the proximal carpal row, distal radius, and ulnar head

The stress distributions of the equivalent stress, minimum principal stress, and maximum principal stress in the proximal carpal row, distal radius, and ulnar head were assessed using contour maps for each flexion–extension angle and radioulnar deviation angle. The mean stress values for each region were extracted for each position, and the percentage changes (%) in relation to the neutral position (0° flexion–extension in the flexion–extension model and 0° radioulnar deviation in the radioulnar deviation model) were statistically compared for each region.

**Figure 3:**
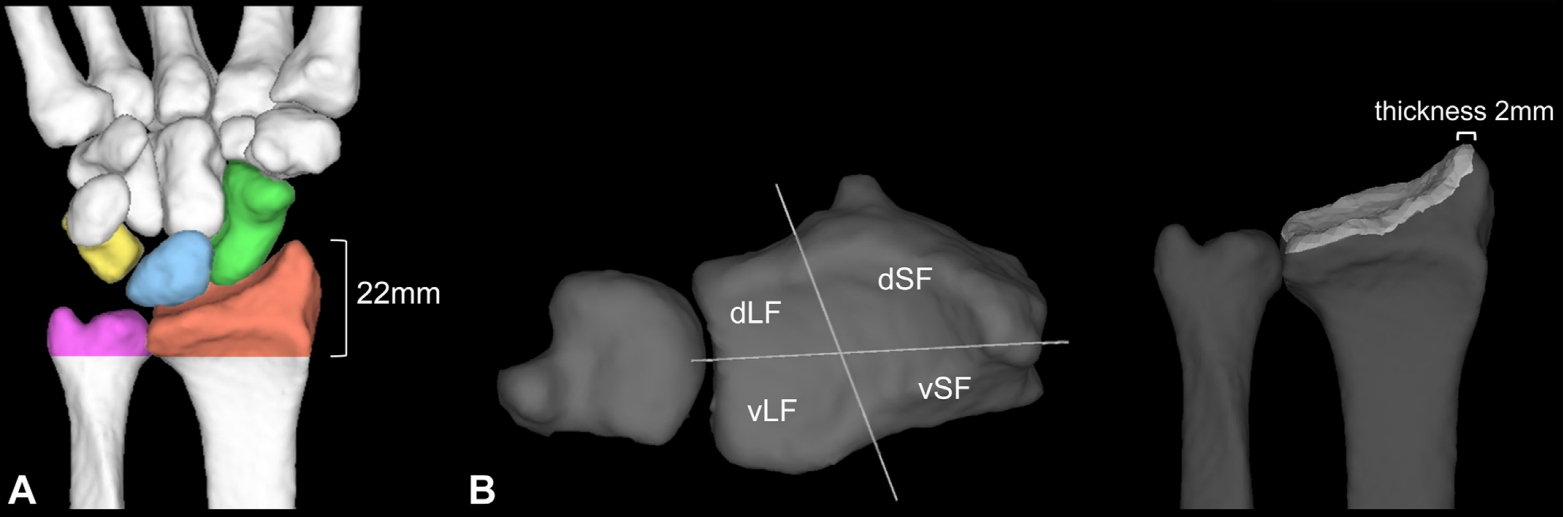
Definition of the ROIs for stress analysis. **A** Definition of ROIs in the distal radius and ulnar head. **B** Regional divisions of the articular surface of the distal radius. The ROI of the distal radius was defined as the region distal to a plane perpendicular to the axis of the radial shaft 22 mm proximal to the radial styloid process. The ROI of the ulnar head was defined as the region distal to the same boundary plane. The articular surface of the distal radius was divided into four ROIs: vLF, dLF, vSF, and dSF. The surface is divided in the radioulnar direction by the ridge between the scaphoid and lunate fossae and dorsopalmarly by a line drawn from the tip of the radial styloid process to the center of the ulnar notch. The analysis region was defined at a depth of 2 mm from the articular surface.

The ROI of the distal radius was defined as the region distal to a plane perpendicular to the axis of the radial shaft 22 mm proximal to the radial styloid process. The ROI of the ulnar head was defined as the region distal to the same boundary plane (Fig. 3A).

#### Intra-articular stress distribution in the distal radius

The distal radius was divided into four ROIs and the percentage (%) of each region’s mean equivalent stress. The stress was calculated in relation to the total stress. The region was divided in the radioulnar direction by the ridge between the scaphoid and lunate fossae and dorsopalmarly by a line drawn from the tip of the radial styloid process to the center of the ulnar notch. These divisions created four ROIs: the volar lunate fossa (vLF), dorsal lunate fossa (dLF), volar scaphoid fossa (vSF), and dorsal scaphoid fossa (dSF). Referring to the study by Bain et al^20^ ROI thickness was set at 2 mm from the articular surface (Fig. 3B).

Statistical comparisons were made for each flexion–extension angle and radioulnar deviation angle between the lunate fossa (vLF + dLF) and scaphoid fossa (vSF + dSF), as well as between the volar (vLF + vSF) and dorsal (dLF + dSF) regions.

### Statistical analysis

Statistical analysis was performed using repeated-measures analysis of variance with Mauchly’s test of sphericity to check for homoscedasticity. When the sphericity assumption was violated, the Greenhouse–Geisser correction was applied. For items showing significant differences, multiple comparisons were performed using Holm’s method. The significance level was set at 5%.

## Results

### Articular surface stress distribution in the flexion–extension models

**Figure 4:**
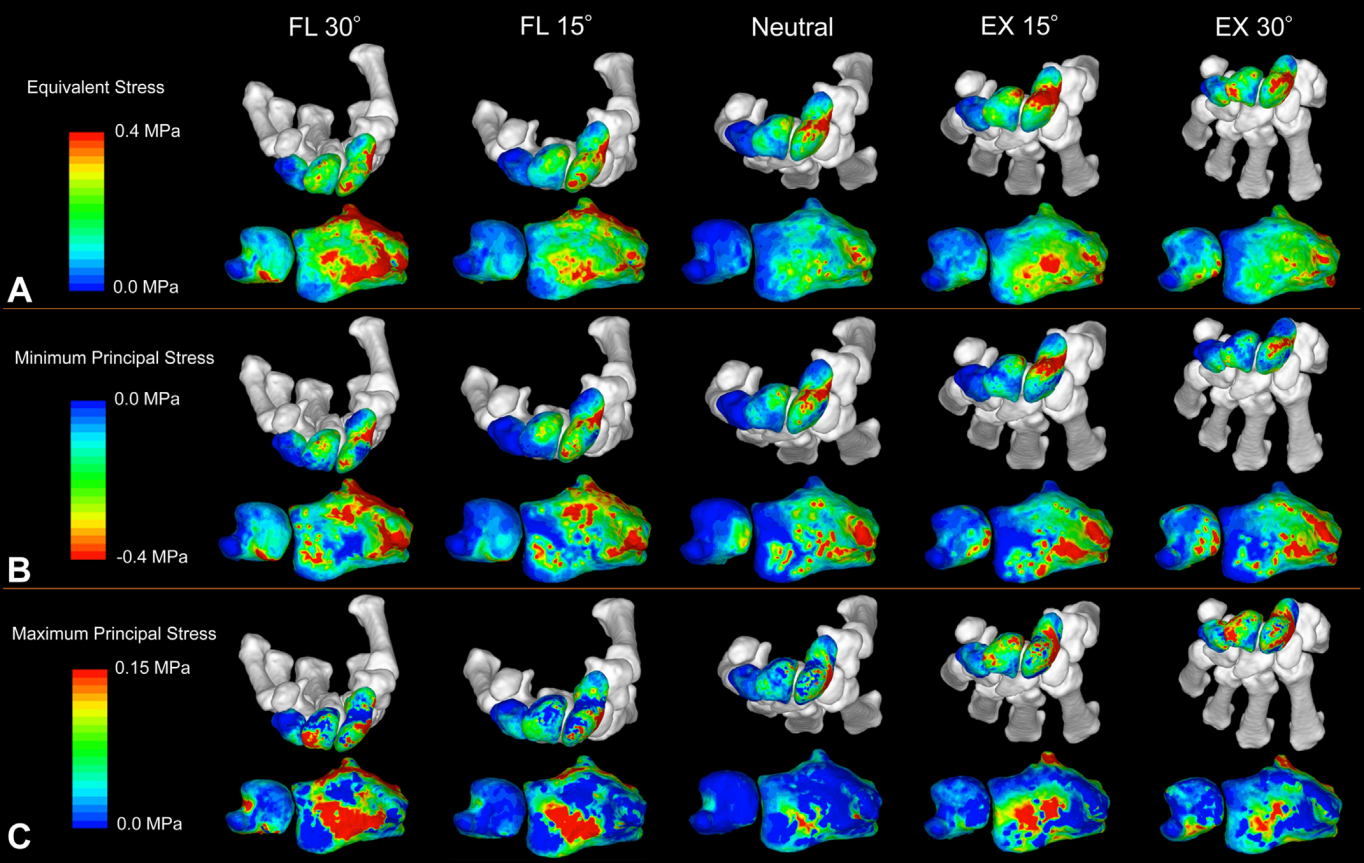
Contour maps of stress distribution in flexion–extension models. **A** Equivalent stress. **B** Minimum principal stress. **C** Maximum principal stress. For the distal radius: the equivalent stress was concentrated on the central articular surface and increased with greater flexion–extension angles. The minimum principal stress was concentrated on the dorsal aspect in flexion and the volar aspect in extension. The maximum principal stress was concentrated in the central distal radius and increased with greater flexion–extension angles. In the ulnar head and triquetrum: equivalent stress and minimum principal stress were concentrated on the volar aspect of the triquetrum and ulnar head in flexion and on the dorsal aspect of both bones in extension. In the scaphoid and lunate: equivalent stress and minimum principal stress were concentrated on the scaphoid waist in extension, whereas stress was concentrated on the dorsal aspect of the scaphoid in flexion. Conversely, in the lunate, stress was concentrated on the dorsal aspect in extension and the volar aspect in flexion. The maximum principal stress in the lunate was concentrated on the nonarticular (dorsal) portion during flexion and shifted to the volar aspect during extension.

The contour maps of stress distributions in the proximal carpal row, distal radius, and ulnar head are shown from a representative specimen (Fig. 4). For the distal radius, the equivalent stress was concentrated on the central articular surface and increased with greater flexion–extension angles. The minimum principal stress was concentrated on the dorsal aspect in flexion and the volar aspect in extension. The maximum principal stress was concentrated on the central distal radius and increased with greater flexion–extension angles.

In the ulnar head and triquetrum, equivalent stress and minimum principal stress were concentrated on the volar aspect of the triquetrum and ulnar head in flexion and on the dorsal aspect of both bones in extension. In the scaphoid and lunate, equivalent stress and minimum principal stress were concentrated on the scaphoid waist during extension, whereas stress was concentrated on the dorsal aspect of the scaphoid in flexion. Conversely, in the lunate, stress was concentrated on the dorsal aspect in extension and the volar aspect in flexion. The maximum principal stress in the lunate was concentrated on the nonarticular (dorsal) portion during flexion and shifted to the volar aspect during extension.

**Figure 5:**
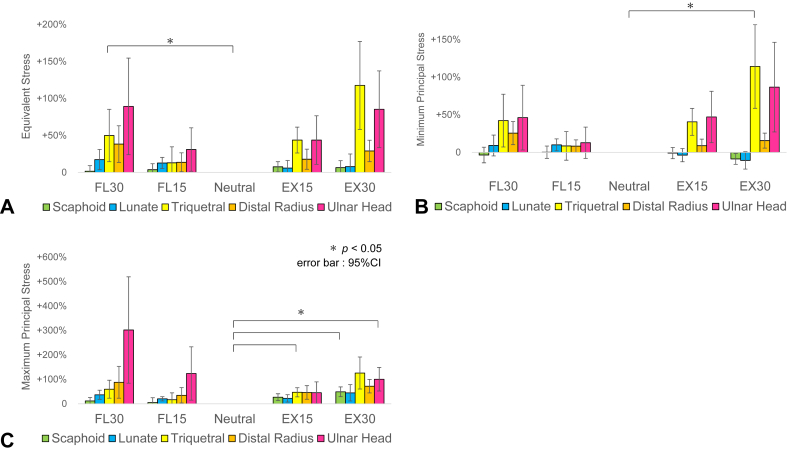
Changes in the stress values for each region in the flexion–extension models. **A** Equivalent stress. **B** Minimum principal stress. **C** Maximum principal stress. The mean stress values of each region in the flexion–extension models were compared with those in the neutral position, and the percentage changes (%) in relation to the neutral position were displayed for each region. The ulnar head and triquetrum increased in all stress measures (equivalent stress, minimum principal stress, and maximum principal stress) during flexion and extension. The lunate and scaphoid showed minimal changes in all stress measurements.

**Figure 6:**
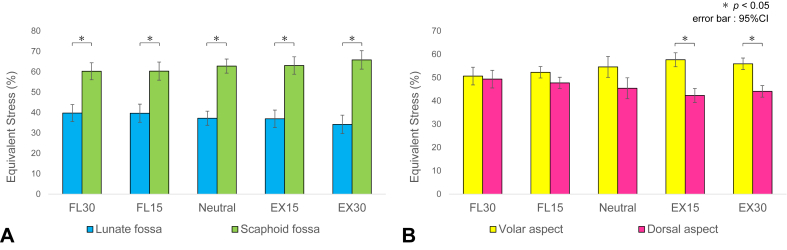
Changes in the stress distribution within the distal radius in flexion–extension models. **A** Analysis of the lunate and scaphoid fossae. **B** Analysis of the volar and dorsal aspects. Percentage distribution of the mean equivalent stress in the four regions of the distal radius relative to the total stress was calculated and displayed for each flexion–extension angle. The comparison of equivalent stress between the lunate fossa (vLF + dLF) and the scaphoid fossa (vSF + dSF) revealed that the scaphoid fossa region was responsible for 60%–66% of the total stress. The comparison between the volar (vLF + vSF) and dorsal (dLF + dSF) regions showed increased stress in the volar aspect during extension and in the dorsal aspect during flexion.

The mean stress values for each region relative to those in the neutral position are shown (Fig. 5). The ulnar head and triquetrum increased in all stress measures (equivalent stress, minimum principal stress, and maximum principal stress) in flexion and extension. The lunate and scaphoid showed minimal changes in all stress measurements. The comparison of equivalent stress in the distal radius between the lunate fossa (vLF + dLF) and the scaphoid fossa (vSF + dSF) revealed that the scaphoid fossa region was responsible for 60% to 66% of the total stress (Fig. 6A). The comparison between the volar (vLF + vSF) and dorsal (dLF + dSF) regions showed increased stress in the volar aspect during extension and in the dorsal aspect during flexion (Fig. 6B).

### Articular surface stress distribution in the radioulnar deviation models

**Figure 7:**
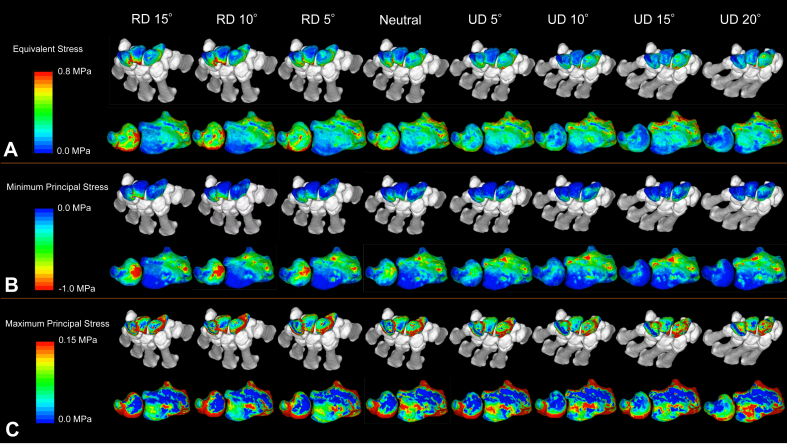
Contour maps of stress distribution in radioulnar deviation models. **A** Equivalent stress. **B** Minimum principal stress. **C** Maximum principal stress. For equivalent stress and minimum principal stress, RD decreased the stress on the lunate fossa of the distal radius while increasing the loads on the ulnar head, triquetrum, and dorsal portion of the lunate (located near the ulnar head). In the UD, the stress in the ulnar head decreased while the stress in the lunate fossa increased.

The contour maps of stress distributions in the proximal carpal row, distal radius, and ulnar head are shown from a representative specimen (Fig. 7). For equivalent stress and minimum principal stress, RD decreased the stress on the lunate fossa of the distal radius while increasing the loads on the ulnar head, triquetrum, and dorsal portion of the lunate (located near the ulnar head). During UD, stress in the ulnar head decreased, whereas stress in the lunate fossa increased.

**Figure 8:**
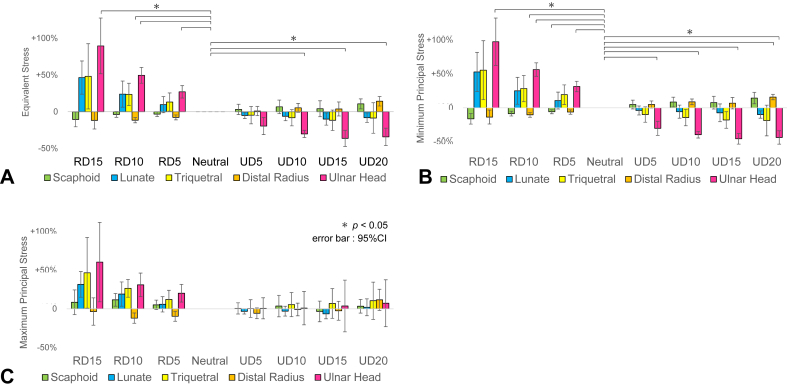
Changes in the stress values for each region in the radioulnar deviation models. **A** Equivalent stress. **B** Minimum principal stress. **C** Maximum principal stress. The mean stress values of each region in the radioulnar deviation models were compared with those of the neutral position, and the percentage changes (%) from the neutral position were displayed for each region. The lunate, triquetrum, and ulnar head showed an increase in equivalent stress and minimum principal stress with increasing RD and decreased stress with increasing UD. The changes in the ulnar head were statistically significant.

The mean stress values for each region relative to those in the neutral position are shown (Fig. 8). The lunate, triquetrum, and ulnar heads showed an increase in equivalent stress and minimum principal stress with increasing RD, and a decrease in stress with increasing UD. Comparison of equivalent stress in the distal radius between the lunate and scaphoid fossae as well as between the volar and dorsal aspects, showed minimal changes in stress distribution (Fig. S1, available online on the Journal’s website at https://www.jhsgo.org).

## Discussion

In this study, the flexion–extension models showed that stress was concentrated on the central articular surface of the distal radius and increased with greater flexion–extension angles. The ulnar head and triquetrum showed increased stress in flexion and extension, whereas the scaphoid and lunate showed minimal changes in stress. In the distal radius, the scaphoid fossa region accounted for 60% to 66% of the total stress. The stress increased on the volar aspect of the distal radius during extension and on the dorsal aspect during flexion. In the radioulnar deviation models, the RD decreased the stress on the lunate fossa and increased the loads on the ulnar head, triquetrum, and dorsal part of the lunate. Conversely, RD decreased stress on the ulnar head and increased stress on the lunate fossa, particularly on the volar aspect.

In the flexion–extension models, stress distribution showed various changes with flexion–extension movement. In the distal radius, flexion induced compressive stress on the dorsal aspect, leading to tensile stress in the central region, whereas extension generated compressive stress on the volar aspect, also resulting in central tensile stress. This tensile stress contributed to the increase in equivalent stress. Rikli et al^2^ reported that after embedding sensors in healthy radiocarpal joints, they found loads of 31 N in the neutral position, 107 N during active flexion, and 197 N during active extension. However, as their sensors could not measure tensile stress, the actual loads were likely higher when considering the tensile stress measured in our study. The scaphoid fossa region sustained greater loads than the lunate fossa region. Previous studies have consistently reported higher load transmission rates in the scaphoid fossa during the neutral position, which aligns with our findings.^4^^,^^21^, ^22^, ^23^, ^24^, ^25^, ^26^ This ratio was maintained throughout the 30° flexion–extension range, suggesting that the scaphoid consistently supports higher loads than the lunate. The lunate showed a stress concentration in its area of contact with the radius, whereas the scaphoid showed different stress distribution patterns. Although the mean stress values of the scaphoid remained unchanged during flexion–extension, stress concentrated at the scaphoid waist during extension. Majima et al^23^ using a 3D rigid-body spring model, reported that in 90° extension, the proximal scaphoid is fixed between the radius and capitate, whereas the distal scaphoid receives force from the trapezium, which can cause fracture of the girdle due to bending stress. In this study, compressive stress on the scaphocapitate articular surface of the scaphoid increased during extension, supporting the hypothesis that the fixation of the scaphoid during extension may lead to waist fractures due to bending stress (Fig. S2, available online on the Journal’s website at https://www.jhsgo.org).

In the radioulnar deviation models, stress distribution changes in the distal radius were less pronounced compared with those in the flexion–extension models. The lunate, triquetrum, and ulnar head exhibited greater stress distribution changes than the distal radius. The equivalent stress and minimum principal stress in the lunate, triquetrum, and ulnar head were concentrated during RD, with statistically significant changes observed in the ulnar head. During RD, the lunate shifted ulnarly, likely causing increased compressive stress through contact with the ulnar head. In the distal radius, minimum principal stress showed minimal changes at all positions, whereas equivalent stress and maximum principal stress were concentrated on the volar aspect of the lunate fossa during UD. This likely occurred because the lunate shifted radially during UD, decreasing its contact area with the ulnar head and increasing its contact with the lunate fossa. Hara et al^4^ used pressure-sensitive conductive rubber sensors in cadaver studies and reported increased load transmission through the ulnar head during RD, which aligns with our findings. The impaction between the lunate and ulnar head reminds us of ulnar impingement syndrome and subsequent lunate bone cysts. Rhee et al^27^ reported that although 10.4% of healthy individuals have lunate cysts, the prevalence increases to 57.6% in patients with ulnar impingement syndrome. Our results indicate that these cysts may develop due to increased contact pressure between the lunate and ulnar head during RD. However, some studies have reported increased ulnar loading during UD, indicating that further research is needed.^5^^,^^28^

This study has several limitations. First, we used a simplified model that did not incorporate soft tissues, such as ligaments and joint capsules. We assumed that within the range of 30° of flexion–extension and 15° of RD to 20° of UD, the ligaments and joint capsules would not experience significant tension; therefore, these structures were replaced with cartilage (Young’s modulus: 10 MPa, Poisson’s ratio: 0.49) to fill all interosseous spaces.^11^^,^^13^ Matsuura et al^11^^,^^29^ validated an FEA model without soft tissues for predicting distal radius fractures by comparing it with studies on fresh and frozen cadavers. Similarly, Yokota et al^30^ demonstrated the validity of a simplified FEA model of the carpal bones, showing results comparable with pressure measurements on fresh-frozen cadavers. Based on these findings, we consider our proposed model valid. Second, the sample size was limited. However, in other subject-specific FEA validation studies, sample sizes of three or fewer are common, with only a few studies using more than three specimens.^13^^,^^14^^,^^31^, ^32^, ^33^, ^34^, ^35^, ^36^, ^37^ Our study included eight specimens, which we believe is sufficient. Third, the cadaver specimens were limited to osteoporotic bones from elderly individuals, and these findings may not be directly applicable to younger patients. This is an inherent limitation of cadaver studies. Nonetheless, we believe that FEA is a valuable tool for evaluating real in vivo stress distributions that cannot be assessed through experiments on fresh-frozen cadavers.

## Declaration of Generative AI and AI-Assisted Technologies in the Writing Process

During the preparation of this work, the authors used Claude 3 for English translation. After using this tool, the authors reviewed and edited the content as needed and takes full responsibility for the content of the publication.

## Conflicts of Interest

No benefits in any form have been received or will be received related directly to this article.
