# Supplementary material for: Mechanical Effects of Wrist Position at the Wrist Joint: A Finite Element Analysis
Source: J Hand Surg Glob Online. 2025 May 23;7(4):100747. doi: 10.1016/j.jhsg.2025.100747 (PMC12151170; doi:10.1016/j.jhsg.2025.100747)
Supplement: Supplementary Figure 1 [file mmc1.pptx]

## Slide 1
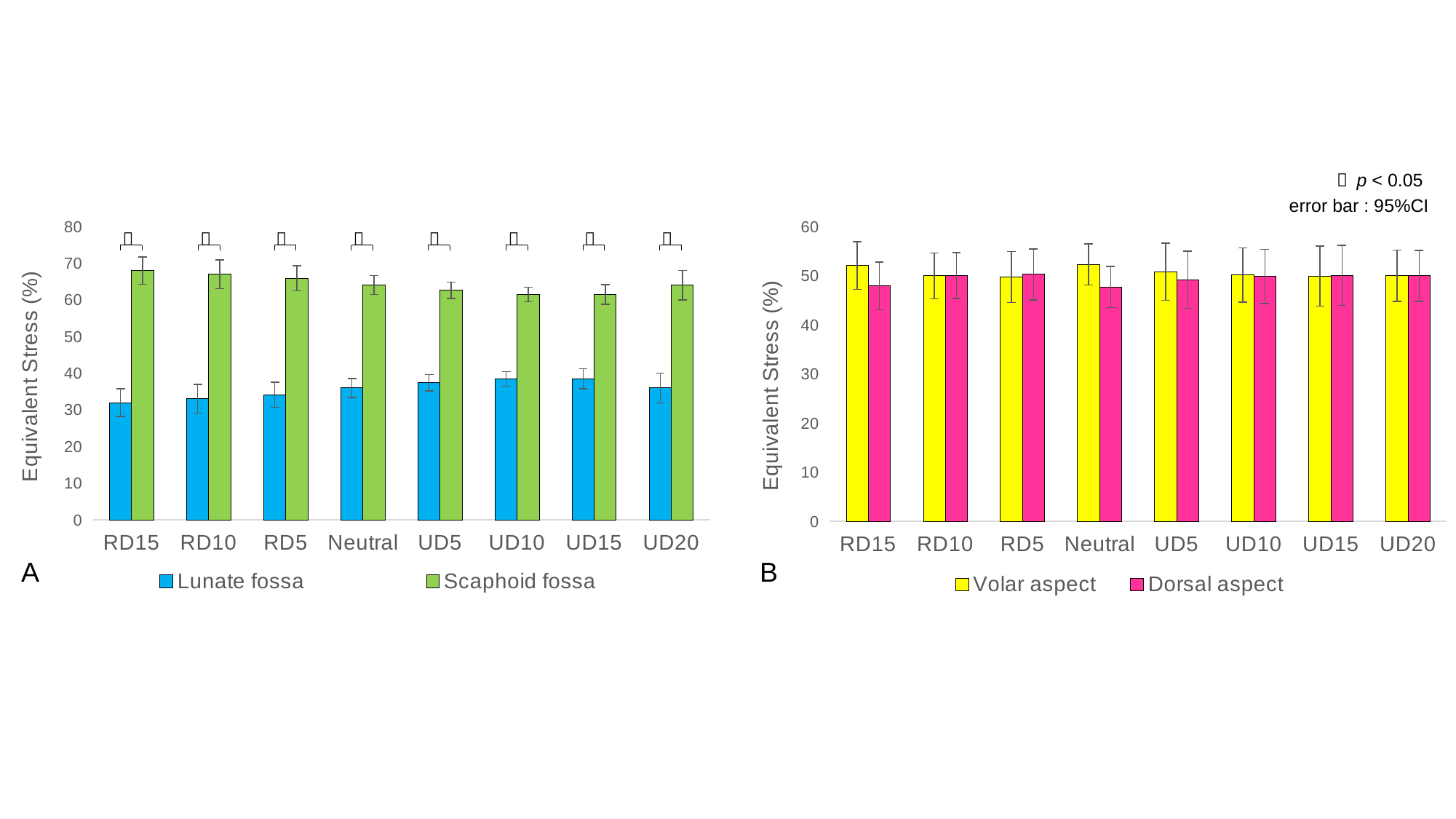

＊ p < 0.05
error bar : 95%CI
### Chart
| Category | Lunate fossa | Scaphoid fossa |
|---|---|---|
| RD15 | 31.984143555258903 | 68.01585644474108 |
| RD10 | 33.0239422331514 | 66.9760577668486 |
| RD5 | 34.10284191522065 | 65.89715808477935 |
| Neutral | 35.96746270774657 | 64.03253729225344 |
| UD5 | 37.381796624426684 | 62.618203375573316 |
| UD10 | 38.48516423399347 | 61.51483576600653 |
| UD15 | 38.52420693932871 | 61.47579306067129 |
| UD20 | 35.9695719784043 | 64.0304280215957 |
### Chart
| Category | Volar aspect | Dorsal aspect |
|---|---|---|
| RD15 | 52.08510842390129 | 47.914891576098704 |
| RD10 | 49.96429964960804 | 50.03570035039196 |
| RD5 | 49.731960855529465 | 50.268039144470535 |
| Neutral | 52.30718224764907 | 47.69281775235093 |
| UD5 | 50.82060430633886 | 49.179395693661135 |
| UD10 | 50.15820483701381 | 49.841795162986195 |
| UD15 | 49.921458370508624 | 50.07854162949138 |
| UD20 | 50.00632886626164 | 49.99367113373837 |＊
＊
＊
＊
＊
＊
＊
＊
A
B
