# Supplementary figures and images for: Mechanical Effects of Wrist Position at the Wrist Joint: A Finite Element Analysis
Source: J Hand Surg Glob Online. 2025 May 23;7(4):100747. doi: 10.1016/j.jhsg.2025.100747 (PMC12151170; doi:10.1016/j.jhsg.2025.100747)

## Slide 1
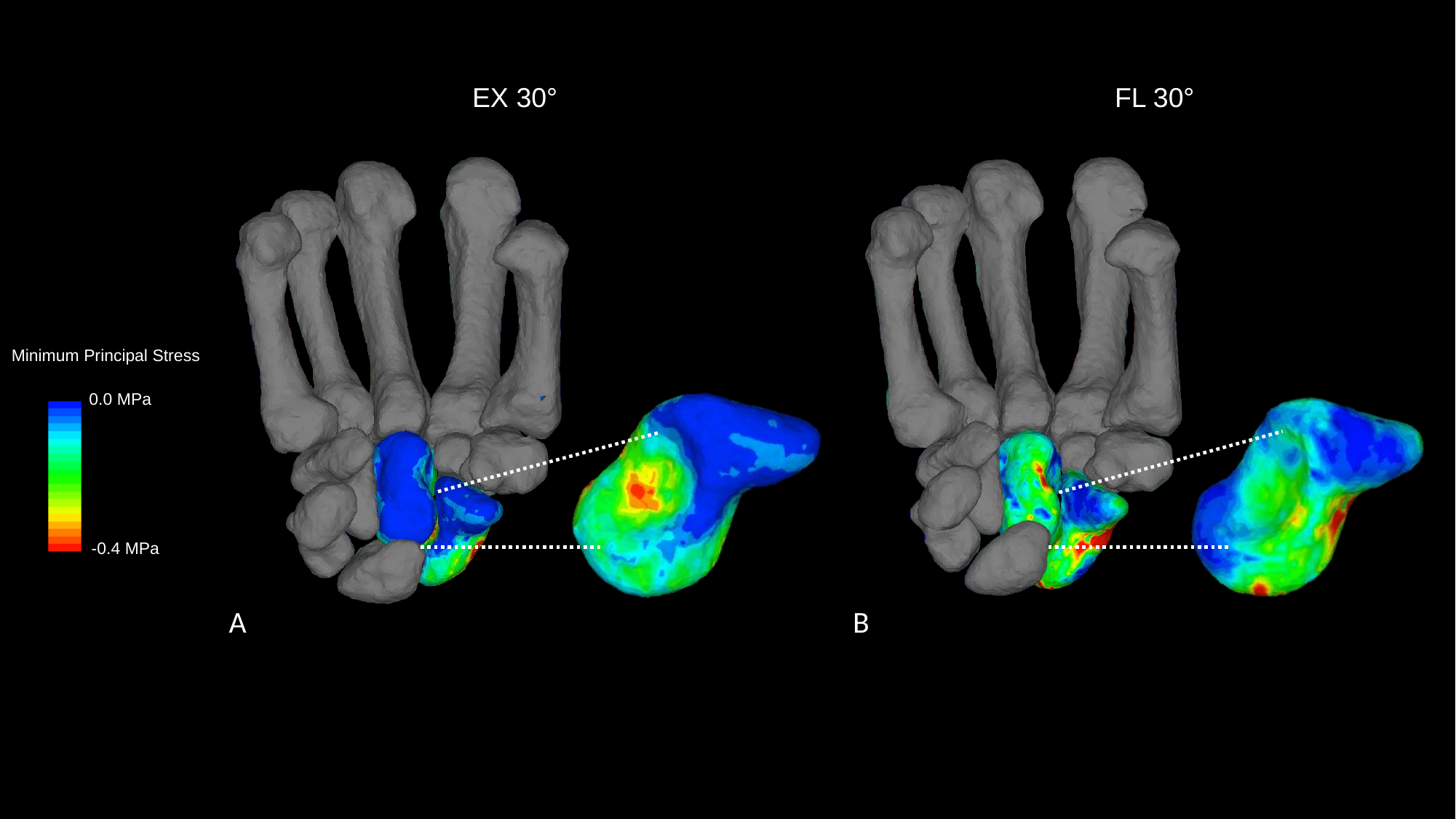

EX 30°
FL 30°
Minimum Principal Stress
0.0 MPa
-0.4 MPa
B
A

Supplement: Supplementary Figure 2 [file mmc2.pptx]
